# Neurological Enhancement Effects of Melatonin against Brain Injury-Induced Oxidative Stress, Neuroinflammation, and Neurodegeneration via AMPK/CREB Signaling

**DOI:** 10.3390/cells8070760

**Published:** 2019-07-21

**Authors:** Shafiq Ur Rehman, Muhammad Ikram, Najeeb Ullah, Sayed Ibrar Alam, Hyun Young Park, Haroon Badshah, Kyonghwan Choe, Myeong Ok Kim

**Affiliations:** 1 Division of Life sciences and Applied Life Science (BK 21plus), College of Natural Science, Gyeongsang National University, Jinju 52828, Korea; 2 Institute of Basic Medical Sciences, Khyber Medical University, Peshawar, Khyber Pakhtunkhwa 25100, Pakistan; 3 Maastricht University Medical Center (MUMC+), School for Mental Health and Neuroscience|Alzheimer Center Limburg, Maastricht 6229ER, The Netherlands

**Keywords:** rmTBI, melatonin, AMPK, CREB, neurodegeneration, amyloid beta, synaptic dysfunction

## Abstract

Oxidative stress and energy imbalance strongly correlate in neurodegenerative diseases. Repeated concussion is becoming a serious public health issue with uncontrollable adverse effects in the human population, which involve cognitive dysfunction and even permanent disability. Here, we demonstrate that traumatic brain injury (TBI) evokes oxidative stress, disrupts brain energy homeostasis, and boosts neuroinflammation, which further contributes to neuronal degeneration and cognitive dysfunction in the mouse brain. We also demonstrate that melatonin (an anti-oxidant agent) treatment exerts neuroprotective effects, while overcoming oxidative stress and energy depletion and reducing neuroinflammation and neurodegeneration. Male C57BL/6N mice were used as a model for repetitive mild traumatic brain injury (rmTBI) and were treated with melatonin. Protein expressions were examined via Western blot analysis, immunofluorescence, and ELISA; meanwhile, behavior analysis was performed through a Morris water maze test, and Y-maze and beam-walking tests. We found elevated oxidative stress, depressed phospho-5′AMP-activated protein kinase (p-AMPK) and phospho- CAMP-response element-binding (p-CREB) levels, and elevated p-NF-κB in rmTBI mouse brains, while melatonin treatment significantly regulated p-AMPK, p-CREB, and p-NF-κB in the rmTBI mouse brain. Furthermore, rmTBI mouse brains showed a deregulated mitochondrial system, abnormal amyloidogenic pathway activation, and cognitive functions which were significantly regulated by melatonin treatment in the mice. These findings provide evidence, for the first time, that rmTBI induces brain energy imbalance and reduces neuronal cell survival, and that melatonin treatment overcomes energy depletion and protects against brain damage via the regulation of p-AMPK/p-CREB signaling pathways in the mouse brain.

## 1. Introduction

Oxidative stress and energy imbalance are implicated in several neurodegenerative diseases, such as Alzheimer’s disease (AD), ischemia, and traumatic brain injury (TBI) [1,2,3,4,5]. Oxidative stress is mainly caused by the excessive accumulation of reactive oxygen species (ROS), which are involved in several cellular abnormalities, such as disturbing the structure and functions of proteins and lipids, DNA damage, and cell death [6]. The relationships between 5′AMP-activated protein kinase (AMPK) and ROS are intricate. In some cells, AMPK is a downstream target of ROS [7]. However, some studies have demonstrated that oxidative stress is an important upstream positive regulator of AMPK. Most importantly, chronic treatment with *N*-acetyl-l-cysteine (NAC, an ROS scavenger) improved the detrimental effects of AMPK and protected against neurodegeneration [8]. Previous studies have reported that the activation of AMPK abolished oxidative stress via the inhibition of reactive oxygen species (ROS)-mediated NAD(P)H oxidase [9]. Another study demonstrated that AMPK activation reduces oxidative stress cyclooxygenase-2 (Cox-2), inducible nitric oxide synthase (iNOS), and uncoupling protein 2 [10]. These studies demonstrated that there exists a positive feedback regulatory mechanism between the activation of AMPK-α1 and ROS. Therefore, the anti-oxidant agent would possibly normalize the APMK level in traumatic brain injury.

5′AMP-activated protein kinase (AMPK) has been identified as an important intracellular energy sensor, deeply involved in energy metabolism [11]. The activation of AMPK suppresses ATP-consuming metabolic pathways, as well as boosting the cellular energy cascade to confer protection against stress-mediated cellular pathophysiology. Several lines of investigations have suggested the potential protective role of AMPK phosphorylation in different models of neurodegenerative conditions [12]. The CAMP-response element-binding (CREB) protein is a well-known pro-survival protein which is controlled by AMPK family member proteins [13]. The CREB protein is a transcription factor, mediating genes closely related to neuronal differentiation, survival, and neurite outgrowth [14,15]. Several kinases have been shown to promote the phosphorylation of CREB at its transcriptional activating site, including the protein kinase A (PKA), protein kinase C, calmodulin kinases, and pp90 ribosomal S6 kinase [16]. The phosphorylation of CREB reduces neuroinflammation by regulating p-NF-κB and further blocking the transcription of inflammatory mediators [17]. Moreover, evidence demonstrates that the phosphorylation of CREB may induce anti-inflammatory cytokines in activated microglia, which are typically involved in microglia de-activation and microglia polarization into the M2 phenotype [18]. 

Traumatic brain injury (TBI) is a major cause of morbidity and mortality, resulting in an unfolding sequence of injury to the central nervous system (CNS), and accounts for serious health problems worldwide. Moderate-to-severe TBI patients suffer from widespread cognitive and behavioral problems, such as executive function, long-term memory impairment, attention, anxiety, and working memory [19,20]. Hallmarks of the secondary insult response in TBI include a depletion of energy [3], reactive oxygen species (ROS) generation, neuroinflammation, and apoptotic cell death, all of which occur immediately following the primary mechanical insult. A recent study also demonstrated that metabolic suppression is associated with the secondary pathology of both pre-clinical and clinical TBI [3,21]. Another study demonstrated that persistent neuroinflammation and disruptions in brain energy metabolism are commonly observed in the brain of TBI and the inhibition of neuroinflammation and regulation of energy homeostasis are associated with an enhancement in cognitive function in the brain [22]. Mild TBI is commonly described in sportspersons involved in sports such as football, boxing, and hockey, as well as war-affected military personnel. It is believed that TBI generates a variety of events and is a risk factor for the development of several neurodegenerative diseases, such as Alzheimer’s disease (AD) [23] and Parkinson’s disease (PD) [24]. Because of multipathological conditions after brain injury, the exact molecular mechanism(s) remain to be fully elucidated.

Melatonin is a hormone derived from tryptophan metabolism that is synthesized and secreted by the pineal gland [25]. Melatonin facilitates neuronal differentiation [26] and also presents antioxidant and antiapoptotic effects [27]. Due to the latter effects, melatonin has been associated with neuroprotective roles [28]. Furthermore, melatonin has been shown to exert neuroprotective effects in experimental models of a wide range of neurological disorders, including stroke [29], an Alzheimer’s disease mouse model [30], and ischemic hypoperfusion [31]. 

Knowledge regarding energy imbalance after repetitive mild traumatic brain injury and, specifically, whether melatonin treatment would overcome the energy imbalance, remains absent. Therefore, we extended our line of investigation and developed an rmTBI mouse model to explore the energy homeostasis and possible regulatory mechanism of melatonin treatment in the mouse brain. Following treatment of rmTBI animals with melatonin, we found that melatonin treatment regulated the energy imbalance and reduced neuroinflammation and brain damage via the regulation of p-AMPK/p-CREB/p-NF-κB signaling in the TBI mouse brain. 

## 2. Material and Methods

### 2.1. Animals and Treatment 

Male C57BL/6N mice (10 weeks, average body weight of 25–30 g) were purchased from (Samtako Bio, Osan, Korea). After arrival, all the animals were acclimatized for 1 week in the animal care center at Gyeongsang National University, South Korea, as previously described [32,33,34]. All the experiments and animal experimental procedures were approved (Approval ID: 125) by the animal ethics committee (IACUC) of the Division of Life Science, Department of Biology, Gyeongsang National University, Republic of South Korea. 

### 2.2. Repetitive Mild Traumatic Brain Injury 

The animals were randomly divided into four groups (*n* = 15): a saline-treated control group (Control), repetitive mild traumatic brain injury (rmTBI) group, repetitive mild traumatic brain injury plus melatonin-treated group (rmTBI plus Mel), and a sham-treated group (Mel). The melatonin-treated group received a daily intraperitoneal injection (i.p) of melatonin (20 mg/kg, daily, i.p). The sham-treated group received a daily i.p injection of melatonin (20mg/kg, daily, i.p) for 7 days without head injury. For the surgical procedures, the mice were anesthetized with Zoletil (0.1 mL/100 g body weight) and Rompun (0.05 mL/100 g body weight) and placed on a stereotaxic frame. Surgery was performed on the animals in a temperature-controlled environment. The skull was exposed by removing the skin (mid-longitudinal incision). The mice were placed on a delicate task wiper (Kimwipe, Kimberly Clark) and manually positioned under a hollow guided perforated tube. TBI was induced by dropping a 58-g weight from a 28-cm height along the cylindrical glass tube on the right frontal side of the head. The mice were subjected to rmTBI (3 times/week). Following the impact, the skin was sutured and the mice were allowed regular heating until fully recovered. The mice were returned to their respective cages. A second injury was performed the next day, the mice were allowed to rest for 1 day, and the third injury was performed on the 4th day after the first injury. The experiments were performed under continuous heating with a heating lamp and monitored visually until full recovery. The saline-treated control followed the same procedures and anesthesia duration on each occasion in order to control for the effects of repeated anesthesia.

### 2.3. Antibodies and Reagents

The following antibodies were used in western blot and immunofluorescence studies: p-AMPK, AMPK p-CREB (Cell Signaling), anti-Nrf-2, anti-PSD-95, anti-Syntaxin, anti-synaptosomal-associated protein 23 (SNAP-23), anti-Caspase 3, anti- PARP-1, anti-Cleaved Caspase-3, Synaptophysin, anti-Bax, anti-Bcl2, anti-TNF-α, anti-IL-1β, anti-p-NF-κB, anti-Iba-1, anti-GFAP, and anti-β-actin, which were all obtained from Santa Cruz Biotechnology (Dallas, TX, USA). Primary antibodies were diluted in 1× TBST (Tris-buffered saline plus Tween) (1:1000), and secondary conjugated anti-mouse horseradish peroxidase (HRP) and conjugated anti-rabbit HRP were diluted 1:10,000 in 1× TBST, all purchased from Promega USA. For confocal microscopic studies, the secondary fluorescent antibodies used were goat anti-mouse and goat anti-rabbit diluted in 1 × 100 phosphate-buffered saline (PBS). 

### 2.4. Immunoblotting

The protein concentrations were quantified by using a Bradford assay (Bio-Rad Protein Assay kit, Bio-Rad Laboratories, CA, USA) as previously described, with modification [35,36,37]. Briefly, the brain tissues were homogenized within PRO-PREP^TM^ solution (iNtRON Biotechnology, Gyeonggi-do, 13202, South Korea) and an equal volume of 20–30 μg of proteins was mixed with 2× Sample Buffer (Invitrogen). Proteins were run on SDS-PAGE and were transferred to polyvinylidene difluoride membranes (PVDF) (Millipore Massachusetts). All the membranes were blocked with 5% skimmed milk and incubated overnight with the primary antibodies at 4 °C. On the next day, membranes were washed with 1xTBST (3 × 8min), followed by incubation with horseradish peroxidase-conjugated secondary antibodies diluted in 1× TBST for 1h, and then washed with 1× TBST (3 × 8min) and developed using enhanced chemiluminescence (ECL) detection reagent (EzWestLumiOne, ATTO, Tokyo, Japan) in a dark room, and the immunoblots bands were obtained using X-ray films. ImageJ software was used for band quantification and the graphs were generated using GraphPad Prism 6 software (San Diego, CA 92108 USA).

### 2.5. Immunofluorescence Staining

The immunohistological analysis was performed as previously described, with modification [38,39]. Briefly, the sections were dried overnight, rehydrated in 0.01 Mm phosphate-buffered saline (PBS), and then rinsed in PBS, treated with proteinase-k, and blocked for 60 min in 5% normal goat serum, before being incubated with the primary antibodies (1–100 ratio) overnight at 4 °C. The slides were washed with 1% PBS, followed by treatment with the respective secondary antibodies Fluorescein isothiocyanate (FITC) and tetramethylrhodamine isothiocyanate (TRITC) labeled for 2 h at room temperature. The slides were then washed and 4′,6-diamidino-2-phenylindole (DAPI) was applied for nucleus detection. The slides were mounted with a fluorescent mounting medium (FluoView FV 1000; Olympus, Tokyo, Japan) and the immunofluorescence images were captured by a confocal scanning microscope (FV1000MPE). The expressions were quantified with ImageJ and the graphs were generated with PRISM6 software.

### 2.6. Reactive Oxygen Species (ROS) Assay

The ROS assay was performed in the TBI mouse cortex homogenates, as previously described, with minor modification [40]. The ROS level was measured by 2,7-dichlorodihydrofluorescein diacetate (DCFH-DA). Briefly, the brain homogenates from the treated and normal mouse groups were taken and diluted (1:20 time) with ice-cold Lock’s buffer solution. The obtained concentration of the brain homogenates and Lock’s buffer was adjusted to 2.5 mg tissue per 500 µL concentration. The final reaction mixture of 1 mL, which was comprised of Locke’s buffer (pH 7.4), brain homogenate (0.2 mL), and 10 mL of DCFH-DA (5 mM), was allowed to incubate for 15–18 min at room temperature. DCFH-DA incorporated into membrane-bound vesicles and the diacetate group was cleaved by esterases. The reaction mixture was maintained for 30 min for further incubation. The DCFH-DA converted to the fluorescent product 2,7-dichlorofluorescein (DCF). The fluorescent product DCF was measured using a spectrofluorometer (excitation at 484 nm and emission at 530 nm). Data are expressed as pmol DCF formed/min/mg protein.

### 2.7. Fluoro-Jade B (FJB) Staining

Fluoro-Jade B (Cat# AG310, EMD Millipore, Temecula, CA 92590, USA) staining was performed as described previously, with minor modification [41,42]. Briefly, the sections were dried overnight and the hippocampus and cortex were selected for histological analyses. On the next day, the slides were washed (2 × 10 min) with PBS and immersed in NaOH (1% *w*/*v*) solution followed by ethanol (80% *v*/*v*) for 5 min. The slides were then kept in ethanol (70% *v*/*v*, 2min), and washed with distilled water (3 times for 1 min). The slides were kept in freshly prepared potassium permanganate (KMNO4) solution (0.06% *w*/*v*, 10 min), followed by washing with distilled water. FJB solution (0.01% *v*/*v*) containing acetic acid (0.1%) was used to stain the slide, which was then washed (3 times for 1 min) with distilled water and kept in an incubator to dry. The slides were covered with glass coverslips using distyrene plasticizer xylene (DPX); a nonfluorescent mounting medium. Confocal laser scanning microscopy (FluoView FV 1000; Olympus, Tokyo, Japan) was used to capture images. ImageJ software was used for quantification of the FJB signals and the graphs were generated with PRISM6 software.

### 2.8. Measurement of Inflammatory Markers Using ELISA

The inflammatory markers in the ipsilateral cortex homogenates were measured in experimental mice 7 days post-rmTBI. The levels of NF-κB (Cat #: KHO037; Thermo Fisher Scientific, Massachusetts, USA) and TNF-α (Cat #: DY410; R&D Systems, Minneapolis, MN, USA) were analyzed using commercially available kits.

### 2.9. Aβ1–42 ELISA Analysis

After homogenization of the treated mouse brain, the levels of Aβ1-42 were analyzed in the cortex and hippocampus homogenates of all four groups 7 days post-rmTBI using a sandwich ELISA kit (Cat#: KHB3442; Thermo Fisher Scientific). The levels of Aβ1–42 were assessed according to the manufacturer’s instructions.

### 2.10. Beam Walking Test

To examine TBI-associated complex motor movements and coordination, a beam walking test was performed as previously described, with modifications [43]. The test was performed at three different time intervals (1, 3, and 7 days) post-rmTBI. Before starting the test, the animals were subjected to training and allowed to complete four trials 3 h before rmTBI. Briefly, the beam was a wooden bar (length: 1200 mm and width: 21 mm) and was placed above the ground. On another end, a black box was placed for animal acclimatization. The mice were allowed to go on the beam to the box and visit it for 60 s. Thereafter, the mice were placed on the beam at a starting distance of 35 cm from the box. The mice were allowed to go to the box and stay there for 60 s. The step was repeated. On the next day, the mice were placed in the box for 60 s and then allowed to go to the box, with the starting point initially at 35 cm, and this was then gradually increased in terms of the distance from the box up to 100 cm. The experiment was repeated three times to cross the beam, with the mice allowed to rest in the box for 1 min. The mean score was calculated from the three runs for each day.

### 2.11. Lipid Per Oxidation (LPO) Assay

Lipid peroxidation (LPO), a useful marker of oxidative stress, is mainly the degradation of lipids that takes place due to oxidative damage. The LPO levels were measured in the brain homogenates from the control saline-treated mice and treated animal groups (*n* = 8 per group). The level of malondialdehyde (MDA), which is a biomarker of LPO, was measured using the thiobarbituric acid reactive substance (TBARS) assay, according to the manufacturer’s instructions (catalog # K739-100; from Biovision Incorporated, Milpitas, CA, USA). Absorbance at 535 and 520 nm was measured using a spectrophotometer. Data are expressed as relative MDA nmol/mg protein. 

### 2.12. Nissl Staining

Nissl staining was performed to analyze the degree of morphological changes in rmTBI and rmTBI plus melatonin-treated groups. Anesthetized mice (Zoletil (0.1 mL/100 g body weight) and Rompun (0.05 mL/100 g body weight) were perfused with saline to wash out the blood and then perfused with ice-cold 4% buffered paraformaldehyde on day 7 post-rmTBI. Brains were removed and fixed with 4% buffered paraformaldehyde at 4 °C, and were subsequently cryopreserved in 20% sucrose for 72 h. The brains were embedded in optimal cutting temperature (OCT) compound and stored at −80 °C for further histological analysis. Coronal sections (14 µm thick) were cut through cryostat (CM 3050C cryostat, Leica Germany) and affixed to gelatin-coated slides. The slides were dried overnight at 37 °C, followed by hydration with a series of graded alcohols (70%, 95%, and 100%) to distilled water, and then 0.5% cresyl violet solution for 10 min. The sections were then dehydrated, cleared in xylene, and coverslipped on a non-fluorescent mounting medium. Histochemical images were taken with a fluorescence light microscope (*n* = 3). The histochemical intensity (number of dead, fragmented, and shrunken neuronal cells) in the cortex/total area of the brain and hippocampus/total area (CA1 and DG region) was measured through ImageJ software. The image background was optimized according to the threshold intensity for all the groups, and the data are expressed as the relative integrated density for the number of dead neuronal cells/section of the all the samples relative to the saline-treated control mouse group.

### 2.13. Assessment of Brain Lesion Volume

The slides containing the brain tissues were selected and stained with cresyl violet. The procedure for Nissl staining is described in the Nissl staining method. The digital photographs of the rmTBI mouse brains and of the melatonin-treated group were captured and analyzed using ImageJ software. The injured areas of the rmTBI and rmTBI plus melatonin groups were carefully outlined and calculated. The lesion volume was obtained by multiplying the sum of the ipsilateral hemisphere area by the distance between the sections [44].

### 2.14. Morris Water Maze (MWM) Test

The Morris water maze (MWM) test was performed as previously described, with modifications [45,46]. The apparatus used for the MWM test consists of a circular tank filled with water. The water was made opaque by adding a non-toxic white ink. A hidden platform (10 m in diameter) was placed 1 cm below the water surface during the training in one quadrant of the tank. The mice were trained to memorize the position of the platform; if the animals failed to find the platform, they were guided to the platform and placed on the hidden platform for 30 s. The training session continued for four days and the latency time was calculated. On the 5th day, the platform was removed, and a probe test was conducted. In the probe trial, the number of crossings, latency to the platform, and time spent in the target quadrant were considered. The data were recorded using a video tracking system (SMART, Panlab Harvard Apparatus, Bioscience Company, Holliston, MA, USA).

### 2.15. Y-Maze Test

The Y-maze apparatus (20 cm high, 50 cm long, and 10 cm in width at the bottom) was made from black-painted wood and used for behavioral analysis [47]. Simply, the mice were placed in the center of the apparatus and allowed to explore it for three 8 min sessions. The arm entries were observed visually. The spontaneous alternation behavior percentage (%) was defined as the successive entry of the mouse into the three arms on overlapping triplet sets. The spontaneous alternation behavior percentage (%) was calculated as successive triplet sets (entries into three different arms consecutively)/total number of arm entries minus 2) × 100. A higher percentage of spontaneous alternation behavior was considered indicative of improved cognitive performance, and vice versa.

### 2.16. Statistical Analysis

The western blot bands were scanned and analyzed through densitometry using the Sigma Gel System (SPSS Inc., Chicago, IL, USA). The data are presented as the mean ± SEM. Statistical significance was assessed by a one-way analysis of variance (ANOVA), followed by post-hoc analysis. The differences are given in the bar graphs. The symbol * represents a significant difference between the control and TBI, and the symbol # represents a significant difference between TBI and TBI plus melatonin-treated groups. Significance was set at *p* < 0.05.

## 3. Results

### 3.1. Melatonin Treatment Ameliorates Oxidative Stress and Regulates the Expression of p-AMPK and Downstream Signaling in TBI Mouse Brains 

**Figure 1:**
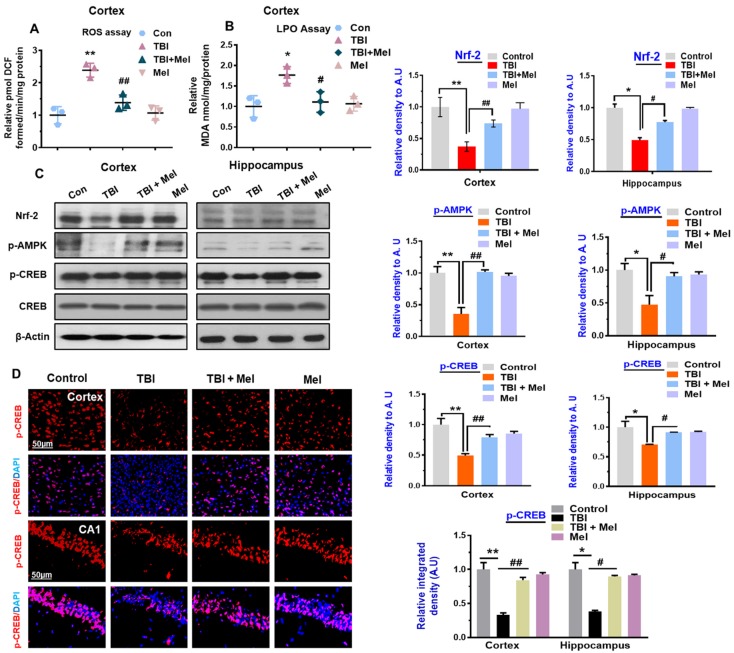
Melatonin treatment reduces oxidative stress and regulates the expression of Nrf-2, p-AMPK, and p-CREB in the repetitive mild traumatic brain injury (rmTBI)-mouse model. (**A**) Representative histogram showing the reactive oxygen species (ROS) level among the treated mice group (*n* = 3). (**B**). Shown is the histogram of the lipid peroxidation among the treated mice group (*n* = 3). (**C**). Western blot analysis and histogram showing expression levels of the Nrf-2, p-AMPK, and p-CREB in the ipsilateral cortex and hippocampus of the rmTBI and rmTBI plus melatonin (Mel)-treated group on day 7. *n* = 10 mice per group. All the values were normalized with beta-actin. Protein bands were quantified with SigmaGel software. (**D**) Confocal microscopic results of p-CREB expressions in the different experimental groups, with respective bar graphs. *n* = 5 mice per group. Magnification 10x. The confocal image’s data was taken from three independent experiments. Differences have been shown in the graphs. The confocal images were analyzed through ImageJ software. The values are expressed as mean ± SEM: One-way ANOVA followed by post-hoc analysis. * *p* < 0.05 and ** *p* < 0.01 represent difference between control and rmTBI, and # *p* < 0.05 and ## *p* < 0.01 represent difference between rmTBI plus Mel-treated group.

**Figure 2:**
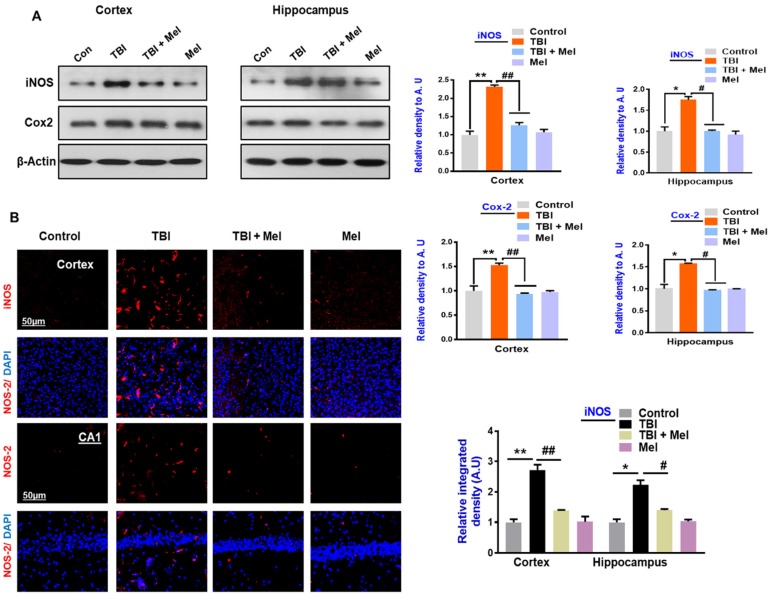
Melatonin regulates the expressions of inducible nitric oxide synthase (iNOS) and cyclooxygenase-2 (Cox-2) in the repetitive mild traumatic brain injury (rmTBI)-mouse model. (**A**) Representative immunoblots and histograms of iNOS and Cox-2 expressions in different experimental groups, with bar graphs. (*n* = 10 mice per group). The beta-actin antibody was used as a loading control. Western blot bands were quantified through SigmaGel software. (**B**) Confocal microscopic results represent iNOS expressions in the different experimental groups, with respective bar graphs. Magnification 10×. *n* = 5 mice per group. Data was taken from three independent experiments. ImageJ software was used for quantitative analysis of the confocal images. The differences have been shown in the graphs. One-way ANOVA followed by post-hoc analysis. * *p* < 0.05 and ** *p* < 0.01 represent difference between control and rmTBI, and # *p* < 0.05 and ## *p* < 0.01 represent difference between rmTBI plus melatonin (Mel)-treated group.

Mounting evidence has reported that the brain is vulnerable to oxidative stress [48]. To examine the relationship between oxidative and energy homeostasis and the possible regulatory mechanism of anti-oxidant melatonin, we analyzed the ROS and LPO levels in the TBI mouse brain. Our results indicated elevated ROS levels and increased MDA contents in rmTBI mouse brains, which were significantly reversed by melatonin treatment (Figure 1A,B). Furthermore, we analyzed the expression of anti-oxidant protein Nrf2 in the rmTBI mouse brain. Our results indicated a reduced level of Nrf2 in the rmTBI-treated mouse brain. However, melatonin treatment significantly reversed the suppressed Nrf2 level in the rmTBI plus Mel-treated mouse brain (Figure 1C), suggesting that the rmTBI mouse brain had elevated oxidative stress from the repetitive concussions and that the treatment with melatonin showed an anti-oxidant capability. Nrf-2 is the regulator of ROS and oxidative stress [49]. Next, we sought to investigate the energy sensor protein AMPK level in the TBI mouse brain and the possible reversal effect of anti-oxidant melatonin in the TBI mouse brain. We observed an imbalance of energy homeostasis on day 7, as revealed by the depressed expression level of p-AMPK in the cortex and hippocampus of the TBI mouse brain compared to the saline-treated mice (Figure 1C). We further examined the expression level of downstream signaling molecules of AMPK in the cortex and hippocampus of the rmTBI-treated mice. p-CREB, the target protein of p-AMPK and a regulator of neuroinflammation [50], was also significantly downregulated in the cortex and hippocampus of the rmTBI mouse brain. Activation of NF-κB has been cited in brain injuries [51]. It was interesting to observe that the treatment with melatonin reversed the energy depletion via regulating the AMPK level, as well as the pro-survival CREB proteins, in the rmTBI plus Mel-treated group, reflecting neuronal injury (Figure 1C). We further validated our results through immunohistological analysis, which indicated a decreased expression level of p-CREB in the cortex and hippocampal CA1 region of the rmTBI mouse group, while the level was significantly elevated in TBI plus Mel-treated mice (Figure 1D). A previous study demonstrated that AMPK activation reduces stress, cyclooxygenase-2 (Cox-2), and inducible nitric oxide synthase (iNOS) [10]. Therefore, we evaluated the expression levels of Cox-2 and iNOS in the rmTBI mouse brain using immunoblot analysis. Our immunoblot results clearly indicated elevated expression levels of Cox-2 and iNOS in the rmTBI mouse brains when compared with the saline-treated mice group. However, interestingly, the expression levels of Cox-2 and iNOS were significantly inhibited upon treatment with melatonin in the rmTBI plus Mel-treated group (Figure 2A). We further validated the expression level of iNOS immunoreactivity in the rmTBI mouse brain. Our confocal microscopy results clearly indicated the significantly increased immunoreactivity of iNOS in the cortex and hippocampal CA1 region of the rmTBI group compared to in saline-treated mouse brains. It was interesting to observe the decreased immunoreactivity of iNOS in the cortex and hippocampus of the melatonin-treated mouse brains, suggesting that anti-oxidant melatonin treatment may inhibit neuroinflammation via the regulation of upstream AMKP levels in the TBI mouse brain (Figure 2B). 

### 3.2. Melatonin Reduced TBI-Induced Neuroinflammation in rmTBI Mouse Brains

**Figure 3:**
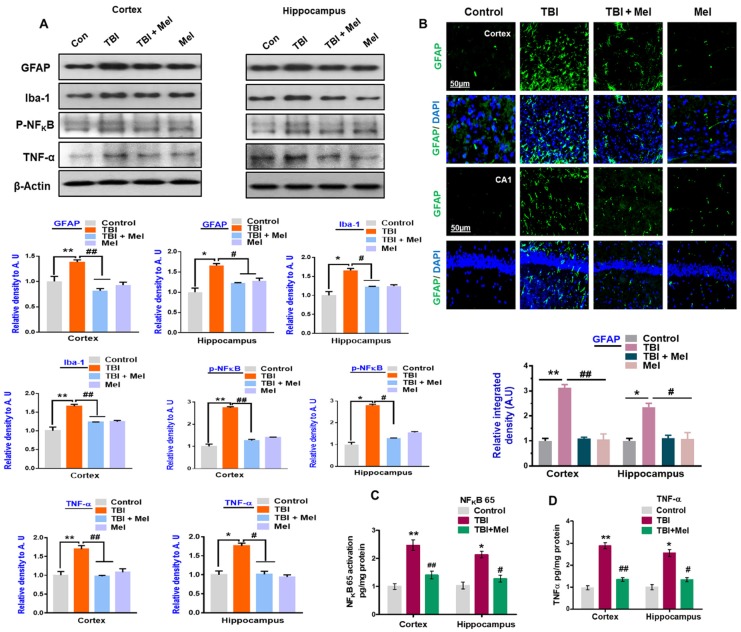
Melatonin regulates gliosis and neuroinflammation in the repetitive mild traumatic brain injury (rmTBI)-mouse model. (**A**) Representative western blots indicating the expression of GFAP (a marker of active astrocytes) and Iba-1(a marker of microglia), p-NF-κB, and tumor necrosis factor-alpha (TNF-α) in rmTBI and rmTBI plus melatonin (Mel)-treated group on day 7. (*n* = 10 mice per group). All the values were normalized with beta-actin. SigmaGel software was used for western blot band quantification. (**B**) Confocal microscopic results of GFAP expressions in the different experimental groups, with respective bar graphs. ImageJ software was used for quantitative analysis of the confocal images. Magnification 10×. *n* = 5 mice per group. Data was taken from three independent experiments. The differences have been shown in the graphs. (**C** and **D**) Histograms represent the NF-κB and TNF-α level in the ipsilateral cortex and hippocampus of the rmTBI and rmTBI plus Mel-treated group through ELISA. The values are expressed as mean ± SEM: One-way ANOVA followed by post-hoc analysis. * *p* < 0.05 and ** *p* < 0.01 represent difference between control and rmTBI, and # *p* < 0.05 and ## *p* < 0.01 represent difference between rmTBI plus Mel-treated group.

Persistent neuroinflammation and imbalance of brain energy metabolism are commonly observed in the TBI-affected brain [22]. Therefore, we examined the inflammatory response in the TBI mouse brains. Our data showed that rmTBI evokes a neuroinflammatory response that generates inflammatory mediators. Our immunoblot analysis showed that there was a marked increase in the expression level of glial fibrillary acidic protein (GFAP); a marker of active astrocytes and ionized calcium binding adaptor moleculeIba-1 (Iba-1); a marker of active microglia in the mouse brain that received rmTBI (Figure 3A). Active astrocytes in the rmTBI mouse brains were further validated through immunofluorescence, which showed an increased number of active astrocytes in the TBI mouse brains (Figure 3B). It was interesting to observe that melatonin treatment significantly reduced the number of active astrocytes and microglia in the rmTBI brains. This observation suggested that a reduction in gliosis might reduce the inflammatory mediators in rmTBI mouse brains. Therefore, we sought to investigate the expression levels of pro-inflammatory mediators in the brains of mice that received rmTBI. As expected, our immunoblot results revealed that the expression levels of p-NF-κB and tumor necrosis factor-alpha (TNF-α) were significantly upregulated in the cortex and hippocampus of the TBI mouse brains compared with the saline-treated mice group. However, treatment with melatonin significantly inhibited the active NF-κB and TNF-α in both ipsilateral sides of the brains of mice that received TBI (Figure 3A). We further strengthened our results: the inflammatory response was confirmed by measuring NF-κB 65 and TNF-α using an ELISA assay in the ipsilateral cortex and hippocampus of the rmTBI mice. The results indicated a significant increase in the levels of NF-κB 65 and TNF-α, which were significantly reversed in the melatonin-treated mice group (Figure 3C,D). Overall, these results support the hypothesis that melatonin may inhibit TBI-induced neuroinflammatory signaling via the regulation of energy homeostasis in the brain.

### 3.3. Melatonin Regulated p-JNK, and Reduced Apoptotic Cell Death and Lesion Volume in an rmTBI Mouse Model

**Figure 4:**
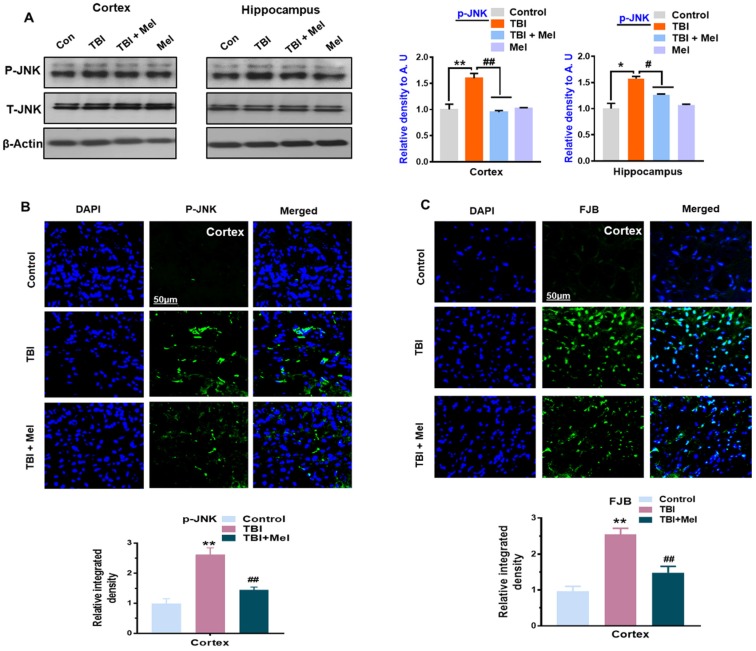
Melatonin modulates the expressions of c-Jun N-terminal kinase (p-JNK) and enhances cell survival in the repetitive mild traumatic brain injury (rmTBI)-mouse model. (**A**) Representative immunoblots and histograms showing p-JNK expressions in the cortex and hippocampus of the experimental groups, with bar graphs. *n* = 10 mice per group. The beta-actin antibody was used as a loading control. Western blot bands were quantified through SigmaGel software. (**B**) Shown are the confocal microscopic results representing P-JNK expressions in the different experimental groups, with respective bar graphs. (**C**) Confocal microscopy results indicating the FJB staining in the cortical region of the rmTBI and rmTBI plus melatonin-treated mice group. *n* = 5 mice per group. Magnification 10×. *n* = 5 mice per group. ImageJ software was used for quantitative analysis of the confocal images. The values were taken from three independent experiments. Protein bands were quantified using SigmaGel software. One-way ANOVA followed by post-hoc analysis. * *p* < 0.05 and ** *p* < 0.01 represent difference between control and rmTBI, and # *p* < 0.05 and ## *p* < 0.01 represent difference between rmTBI plus melatonin (Mel)-treated group.

**Figure 5:**
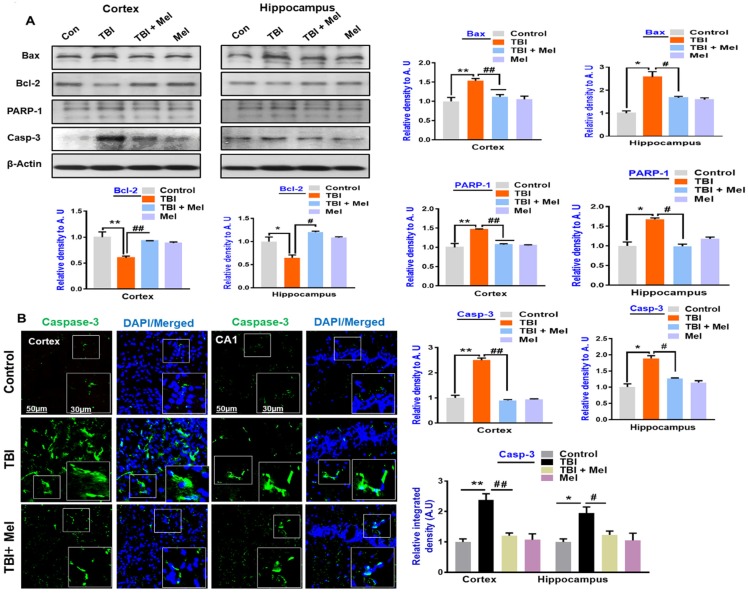

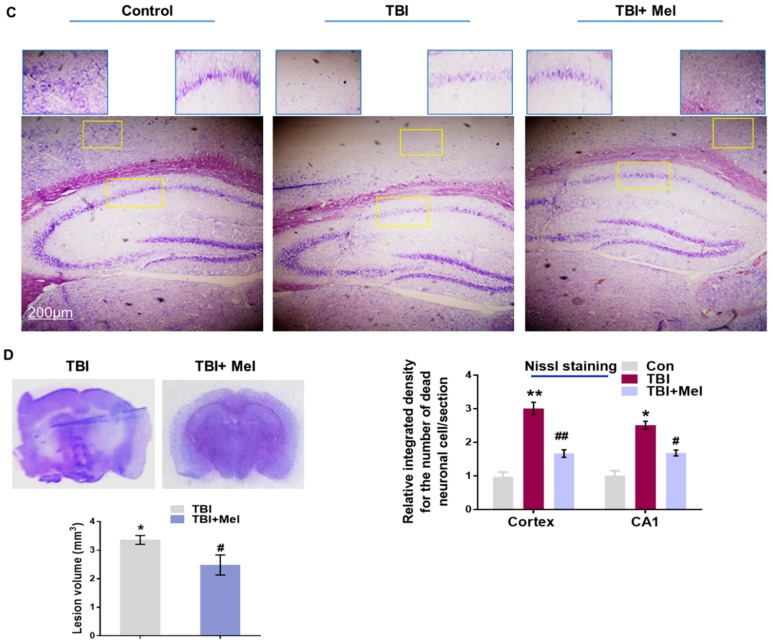
Melatonin rescues the mice brain against the apoptotic cell death associated with repetitive mild traumatic brain injury (rmTBI). (**A**) Western blot analysis and histograms showing the mitochondrial apoptotic signaling markers such as Bax, Caspase-3, PARP-1, and Bcl-2 among the treated mice groups. *n* = 10 mice per group. Each membrane of the respective band was probed with β-actin. β-Actin was used as a loading control. (**B**) Confocal microscopy images represent the immunoreactivity of caspase-3 in the cortex and hippocampal CA1 region of rmTBI and rmTBI plus melatonin-treated mice group. *n* = 5 mice per group. (**C**) Representative photomicrograph of Nissl staining in the cortices and CA1 regions of the mice brain that received rmTBI and rmTBI plus melatonin treatment. (**D**) Representative images of cresyl violet-stained coronal brain sections from rmTBI and rmTBI plus melatonin-treated group. The images were quantified using ImageJ software. Magnification, 10×. The values are expressed as mean ± SEM. One-way ANOVA followed by post-hoc analysis * *p* < 0.05 and ** *p* < 0.01 represent difference between control and rmTBI, and # *p* < 0.05 and ## *p* < 0.01 represent difference between rmTBI plus melatonin (Mel)-treated group.

Previously, it has been reported that TBI causes apoptotic cell death and neurodegeneration [52], and c-Jun N-terminal kinase (JNK), a stress-activated protein kinase was reported to play a critical role in mediating neuronal cell loss and apoptotic neurodegeneration [44]. Here, we analyzed the effects of melatonin on the phosphorylation of c-Jun N-terminal kinase (p-JNK). According to our western blot and immunohistological results, there was an elevated expression of p-JNK in the TBI-induced mouse brains, which was significantly regulated in the melatonin-treated group (Figure 4A,B). Next, we examined TBI-induced apoptotic neurodegeneration. Our western blot findings showed an elevated expression of cleaved caspase-3, Bax, and PAPR1, and reduced expression of Bcl2 in the TBI mouse hippocampi and cortices. It was interesting to find that melatonin reversed the elevated expression levels of these apoptotic markers in the melatonin-treated TBI mice group (Figure 5A). The immunoblot results were further confirmed by immunofluorescence results, showing a marked increase in the expression of Caspase-3 in the TBI mouse brains. Interestingly, the expression was significantly downregulated in the melatonin-treated group (Figure 5B). Furthermore, FJB staining was performed, which indicated an increase in FJB^+^ cells in the TBI cortex compared to the saline-treated mice group, indicating an increasing number of dead neuronal cells. However, FJB^+^ cells were reduced in the melatonin-treated mice group (Figure 4C), supporting the notion that melatonin significantly regulated the expression of apoptotic markers in the TBI plus melatonin-treated group.

In accordance with the FJB results, the Nissl staining results further confirmed that after rmTBI, the number of apoptotic and degenerated neurons was significantly higher in the rmTBI mouse brains compared to the saline-treated control mice group. However, the number of degenerated neuronal cells (damaged, fragmented, or shrunken neuronal cells) was significantly reduced in the melatonin-treated mice group (Figure 5C). Furthermore, the lesion volume was measured in cresyl violet-stained brain sections after rmTBI on day 7. The results showed a significant increase in the lesion volume. However, melatonin treatment condensed the lesion volume in the rmTBI plus melatonin-treated group compared to the rmTBI alone group (Figure 5D).

### 3.4. Melatonin Ameliorated TBI-Induced AD-Like Pathological Changes in the Mouse Brain 

**Figure 6:**
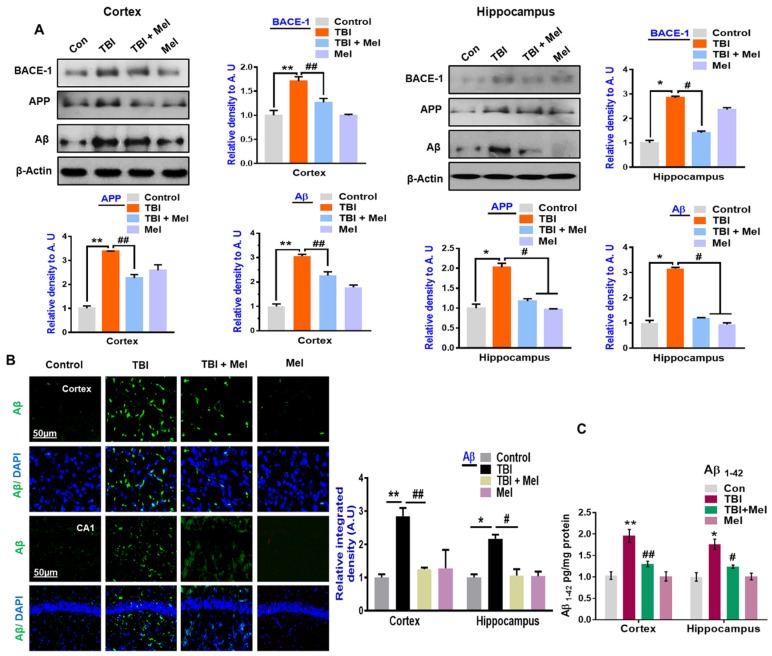
Melatonin inhibits Alzheimer’s disease (AD)-like pathological changes in the traumatic brain injury (TBI)-induced mouse brain. (**A**) Showing the Western blot results of β-APP-cleaving enzyme-1 (BACE-1), amyloid precursor protein (APP), and Aβ in the cortex and hippocampus of the experimental groups, with respective bar graphs. Magnification, 10×. *n* = 10 mice per group. Beta-actin was used as a loading control. (**B**) Confocal microscopic results of Aβ expressions in the different experimental groups, with the respective bar graph. Magnification, 10×. *n* = 5 mice per group. The images were quantified using ImageJ software. (**C**) Representative histogram showing the level of Aβ1-42 in the ipsilateral cortex and hippocampus among the treated mice groups. The differences have been shown in the graphs. The values are expressed as mean ± SEM. One-way ANOVA followed by post-hoc analysis. * *p* < 0.05 and ** *p* < 0.01 represent difference between control and repetitive mild traumatic brain injury (rmTBI), and # *p* < 0.05 and ## *p* < 0.01 represent difference between rmTBI plus melatonin (Mel)-treated group.

Brain injuries are a risk factor for Alzheimer’s disease in the later stage, with no known mechanism [23]. Therefore, we examined the AD-markers in the mouse brain following rmTBI, on day 7. Our western blot results indicated a marked increase in the expression of AD-like markers, such as β-APP-cleaving enzyme-1 (BACE-1), amyloid precursor protein (APP), and Aβ, in the TBI mouse brains (Figure 6A). Similarly, the immunofluorescence results of Aβ also supported our Western blot results, indicating the increased immunoreactivity of Aβ in the cortex and hippocampus of the rmTBI mouse brain (Figure 6B). Interestingly, treatment with melatonin significantly reduced the expression levels of BACE-1, APP, and Aβ in the rmTBI plus Mel-treated group. Furthermore, the more toxic form of Aβ (Aβ_1-42_) was measured using an Aβ_1-42_-specific ELISA assay. We found an elevated level of Aβ_1-42_ in the cortex and hippocampus of the rmTBI-treated mice group compared to the saline-treated group. On the other hand, the elevated level of Aβ_1-42_ was significantly reduced in the cortex and hippocampus of the melatonin-treated group (Figure 6C), suggesting possible neuroprotective effects against TBI-induced neurodegenerative conditions, such as AD-like pathological illnesses in the brain.

### 3.5. Melatonin Rescued Synaptic Protein Loss in the rmTBI Mouse Model

**Figure 7:**
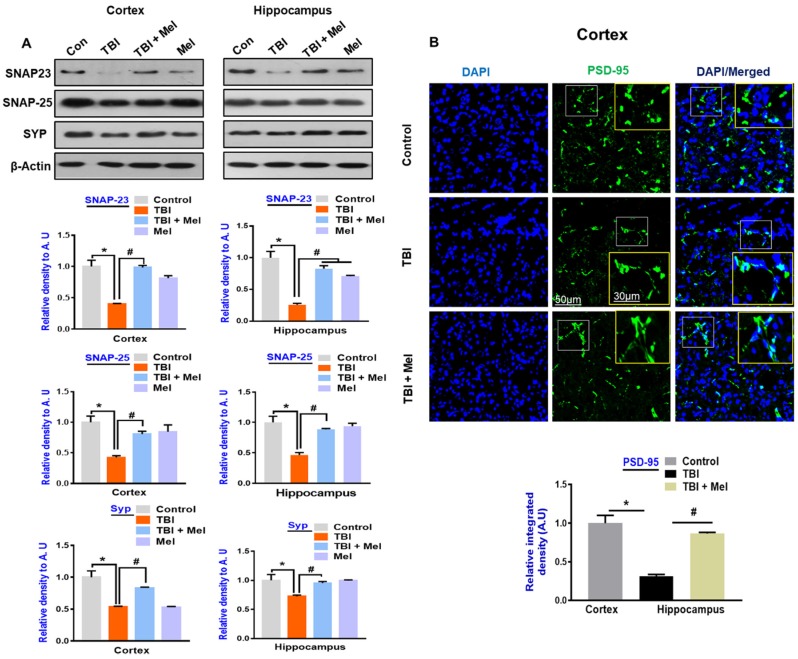
Melatonin restores the depressed expression of synaptic markers in the traumatic brain injury (TBI)-induced mouse brain. (**A**) Western blot results of SNAP-23, SNAP-25, and Synaptophysin in the ipsilateral cortex and hippocampus of the experimental groups, with respective bar graphs. (*n* = 10 mice per group). Beta-actin was used as a loading control. SigmaGel software was used to analyze the data. (**B**) Confocal microscopic results of postsynaptic density protein-95 (PSD-95) expressions in the different experimental groups, with a respective bar graph. Magnification, 10×. *n* = 5 mice per group. Values were taken from three different experiments. The values are expressed as mean ± SEM. One-way ANOVA followed by post-hoc analysis. * *p* < 0.05 and ** *p* < 0.01 represent difference between control and repetitive mild traumatic brain injury (rmTBI), and # *p* < 0.05 and ## *p* < 0.01 represent difference between rmTBI plus melatonin (Mel)-treated group.

Brain injury results in severe damage to the neuronal cell, which results in a deregulated synaptic protein level, thus causing memory problems [53]. We further validated that rmTBI results in synaptic proteins loss, as revealed by the depressed expression of the synaptic proteins, such as SNAP-23, SNAP-25, and Synaptophysin, in the cortices and hippocampi of the rmTBI mouse group (Figure 7A). Interestingly, these markers were significantly upregulated in the melatonin-treated group. Furthermore, the confocal microscopy results also supported the notion that melatonin regulates synaptic proteins levels, as revealed by the reduced expression of postsynaptic density protein-95 (PSD-95) in the brains of the rmTBI plus melatonin-treated group (Figure 7B).

### 3.6. Melatonin Rescued Memory Dysfunction in a rmTBI Mouse Model

**Figure 8:**
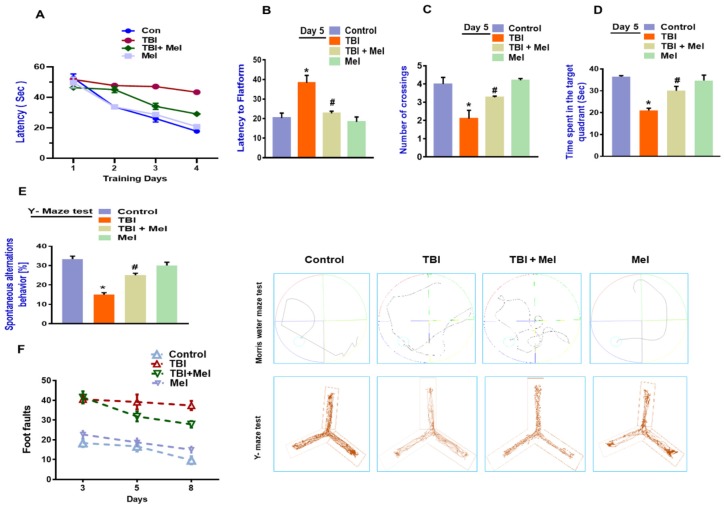
Melatonin improved hippocampal-dependent memory impairment in the mouse model of traumatic brain injury (TBI). (**A**) Representation of the escape latency during training days. (**B**) Histogram showing the escape latency during the probe test. (**C**) Histogram showing the number of crossings during the probe test. (**D**) The histogram represents the time spent in the target quadrant. (**E**) Histogram showing the spontaneous alternations behavior percentage (%) in the y-maze test. *n* = 15 mice per group. (**F**). Representative histogram showing the number of foot faults in the treated group. The values are expressed as mean ± SEM. The differences have been shown in the graphs. The values are expressed as mean ± standard deviation: One-way ANOVA followed by post-hoc analysis. * *p* < 0.05 and ** *p* < 0.01 represent difference between control and repetitive mild traumatic brain injury (rmTBI), and # *p* < 0.05 and ## *p* < 0.01 represent difference between rmTBI plus melatonin (Mel)-treated group.

Synaptic dysfunction has been shown to be responsible for memory and behavioral changes. Both pre-clinical and clinical studies have shown that TBI leads to cognitive deficits [53]. Therefore, we analyzed cognitive abnormalities via examining behavioral performance. Our results demonstrated that rmTBI mice showed memory problems, as shown in the MWM test. Our results indicated that an increased latency time was required to find the hidden platform compared to the saline-treated mice group, indicating spatial memory deficits. However, the rmTBI mice which were treated with melatonin showed a significant reduction in latency time (Figure 8A). Similarly, on the 5th day during the probe test, the latency time required to find the hidden platform was significantly higher compared to the control group (Figure 8B). However, a significant reduction in the latency time was seen in the TBI plus Mel-treated group. Similarly, the number of crossings over the platform was significantly reduced in the rmTBI mice group compared to the saline-treated mice group; however, significant reversal effects were observed, with an increased number of platform crossings suggesting an improved memory performance (Figure 8C). In the case of time spent in the target, there was a significant improvement in the total time spent in the target quadrant in the melatonin-treated mice group compared to the rmTBI group (Figure 8D). In the Y-maze test, the alternation behaviors percentage (%) was significantly reduced in the TBI group but was significantly regulated in the melatonin-treated group (Figure 8D). Furthermore, there was a marked difference in the beam walking test among the treated groups. However, melatonin treatment significantly normalized the difference, showing improved motor function abnormalities after rmTBI (Figure 8F). The overall findings show that melatonin may have neuroprotective outcomes via the regulation of oxidative stress-mediated energy imbalance and downstream signaling after brain injury.

## 4. Discussion

Oxidative stress, disrupted energy homeostasis, neuroinflammation, and apoptosis are commonly seen in the brain after TBI, as well as in other brain diseases [22], which, in turn, can compromise the normal cellular function of the brain [54]. Oxidative stress has been considered the earliest pathological event in several diseases [55]. More specifically, the cortex and hippocampus of the brain are more susceptible to oxidative stress, which ultimately results in memory problems [56]. AMPK senses and regulates energy homeostasis in the brain and its activation promotes anti-inflammatory responses and acts as an antioxidant regulator [57]. Therefore, the pharmacological activation of AMPK may have a disease-modifying latent to treat rmTBI and rmTBI-like conditions in the brain. We hypothesized that anti-oxidant melatonin has the ability to overcome AMPK-dependent neurodegenerative conditions in the rmTBI mouse brain, as AMPK is the downstream target of ROS. Therefore, we sought to investigate oxidative stress and cellular energy imbalance following rmTBI in mouse brains and the regulatory effects of energy homeostasis via anti-oxidant melatonin in TBI mouse brains. Our ROS and LPO assays clearly indicated elevated oxidative stress in the mouse brain following TBI. Furthermore, the antioxidant Nrf-2 was seen to be down-regulated through our western blot results in the brains of the mice that received TBI. However, it was interesting to show that the level of oxidative stress was reversed in the brains of mice that received melatonin treatment, as revealed by the low levels of DCF and MDA contents, as well as the elevated expression of anti-oxidant Nrf-2. Furthermore, we examined the AMPK level in the TBI mouse brain. Consistent with the previous results [22], our immunoblot and immunohistological analyses strongly suggested that the level of AMPK was significantly reduced in the ipsilateral cortex and hippocampus of the rmTBI mouse brain. However, treatment with melatonin for 7 days significantly regulated the level of AMPK in the mouse brain. A recent study demonstrated that AMPK can phosphorylate the CREB family proteins and enhance CREB-dependent transcription [58]. A previous study also indicated a depressed level of CREB following brain injury [59]. CREB phosphorylation stimulates the transcription of genes that are required for cell survival [60].

We extended our line of investigation and explored the depressed level of p-CREB in the ipsilateral cortex and hippocampus of the rmTBI mouse brain. It was interesting to show that melatonin treatment reversed the effects and elevated the level of p-CREB in the above-mentioned regions of mouse brains. Peixoto and his coworkers [61] demonstrated the role of AMPK in cellular death and inflammation. Similarly, we found active astrocytes and microglia and elevated levels of inflammatory mediators such as NF-κB, TNF-α, iNOS, and Cox-2 in the ipsilateral side of the brains of mice that received rmTBI activation. A previous investigation reported chronic neuroinflammation following brain injury [44]. Deregulated brain energy metabolism and persistent neuroinflammation are commonly observed in the brain after injury [22]. It was interesting to observe that melatonin treatment significantly regulated the brain energy balance and inhibited the inflammatory response in the brains of mice that received TBI, supporting its encouraging role against TBI-induced neurodegenerative conditions. Our results are consistent with previous reports demonstrating that melatonin treatment lowers inflammation in animal models [62]. JNK is activated in response to stress insult and regulates several important physiological processes [63], and has also been reported in TBI [44]. It is well-known that MAP kinases regulate several physiological functions; however, their activation leads to pro-inflammatory cytokine production, as well as cell death [64]. Our data also showed an increased expression of active JNK in the TBI mouse brain, which was significantly regulated by treatment with melatonin. Previous studies reported the protective effects of melatonin against apoptotic cells by regulating the expression of JNK [30]. 

Moreover, Jun and his coworker [65] reported that the activation of AMPK ameliorated spinal cord injury-induced neuronal apoptosis. Similarly, our results also showed TBI-induced neuronal apoptosis, which may be due to deregulated energy balance. However, melatonin treatment markedly regulated neuronal apoptosis while reducing the expression levels of Bax, cleaved caspase-3, cleaved PARP-1, and elevated Bcl-2 protein level in the TBI mouse brains. Similarly, it has been shown that melatonin treatment regulates the mitochondrial system and reduces neuronal apoptosis [66]. Furthermore, the Nissl staining results showed a marked increase in the death of neuronal cells and an enlarged lesion volume in rmTBI cortices and these effects were significantly reversed by treatment with melatonin. Several lines of investigation have reported Aβ generation and deposition in the brain following brain injury [67]. An elevated level of Aβ_42_ was examined in the cerebrospinal fluid of head trauma patients [68]. We explored the rmTBI-induced increased level of Aβ in the mouse brain following rmTBI in a mouse model. Our data showed a significant increase in the expression of Aβ, Aβ-generating enzyme, β-APP-cleaving enzyme-1 (BACE1), and amyloid precursor protein (APP) following rmTBI in the mouse brains. All these AD markers were significantly regulated in the melatonin-treated mouse brain. Previous studies reported that the activation of AMPK reduced the amyloidogenic pathway in the mouse brain [69]. Another study reported the critical role of AMPK in the aberrant processing of APP, and also that the activation of AMPK downregulates Aβ production [70], suggesting the possible involvement of AMPK with the amyloidogenic pathway following rmTBI. In the present study, our data showed that melatonin treatment regulated AMPK levels and reduced the amyloidogenic pathway in the mouse brains that received rmTBI and melatonin treatment. 

**Figure 9:**
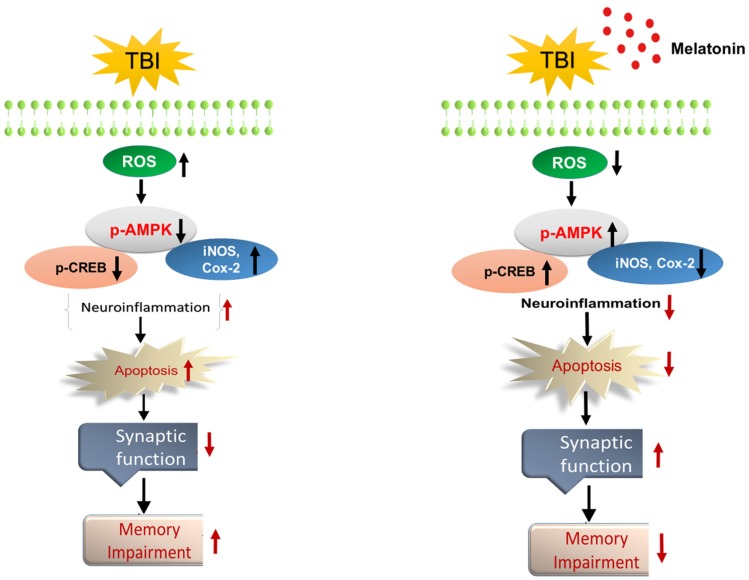
The schematic diagram represents the mechanism of melatonin neuroprotection in mouse brains following traumatic brain injury (TBI). The schematic representation showing that melatonin treatment for 7 days reduced oxidative stress and the AMP-activated protein kinase/CAMP-response element-binding (AMPK/CREB) level, inhibited neuroinflammation and neuronal apoptosis, and improved memory functions via regulation of the AMPK/CREB signaling pathway after TBI.

Several lines of investigations have reported the protective effects of melatonin against Aβ toxicity in the brain and in cell lines [71]. Both pre-clinical and clinical studies have revealed that TBI is associated with increased synaptic loss and cognitive problems [72,73]. Therefore, we sought to investigate the synaptic marker levels and memory problems in our model of rmTBI. It was interesting to show that rmTBI significantly depressed the synaptic protein levels and deregulated cognitive function, as indicated in the MWM and Y-Maze tests. Our MWM test showed an increase in the escape latency time, with a lower number of crossings and less time spent in the target quadrant, indicating a weak memory performance. However, treatment with melatonin overturned the effects, increased the levels of synaptic proteins and regulated cognitive impairments in rmTBI mice. Furthermore, our Y-maze results showed a decrease in spontaneous alternation behavior (%), which indicates spatial working memory problems in the TBI mouse brain. However, we observed an increase in spontaneous alternation behavior (%) in the TBI mice that received melatonin treatment. Furthermore, our results indicated that TBI induces motor neuronal dysfunctions, as was evident in the beam walking test. Importantly, melatonin treatment significantly reversed the motor function deficits in the rmTBI plus melatonin-treated group. This result indicates that treatment with melatonin significantly restored cognitive problems in TBI mice. The overall neuroprotective mechanism of melatonin against TBI is summarized in Figure 9.

## 5. Conclusions

In conclusion, these data provide momentous preclinical evidence that rmTBI induces oxidative stress and neuroinflammation and disrupts energy homeostasis in the mouse brain via deregulated Nrf-2 AMPK/p-CREB and iNOS signaling in the brain. This study also provides evidence that rmTBI is associated with increased neuronal apoptosis, an elevated amyloidogenic pathway, and memory impairment in the mice brain. However, treatment with melatonin efficiently regulates the energy imbalance and reduces neuronal apoptosis, neuroinflammation, the amyloidogenic pathway, and cognitive deficits via Nrf-2/AMPK/p-CREB and iNOS signaling pathways in the mouse brain. Our findings suggest that treatment with melatonin could be a disease-modifying therapeutic strategy to treat TBI-induced neurodegenerative conditions in the brain.
